# Calibrating SANS data for instrument geometry and pixel sensitivity effects: access to an extended *Q* range

**DOI:** 10.1107/S1600576717011463

**Published:** 2017-09-14

**Authors:** Lukas Karge, Ralph Gilles, Sebastian Busch

**Affiliations:** a Heinz Maier-Leibnitz Zentrum (MLZ), Technische Universität München, Lichtenbergstrasse 1, 85747 Garching bei München, Germany; b German Engineering Materials Science Centre (GEMS) at Heinz Maier-Leibnitz Zentrum (MLZ), Helmholtz-Zentrum Geesthacht, Lichtenbergstrasse 1, 85747 Garching bei München, Germany

**Keywords:** small-angle neutron scattering, data reduction, calibration, corrections

## Abstract

An improved data-reduction procedure is proposed and demonstrated for small-angle neutron scattering (SANS) measurements. Its main feature is the correction of geometry- and wavelength-dependent intensity variations on the detector in a separate step from the different pixel sensitivities: the geometric and wavelength effects can be corrected analytically, while pixel sensitivities have to be calibrated to a reference measurement. The geometric effects are treated for position-sensitive ^3^He proportional counter tubes, where they are anisotropic owing to the cylindrical geometry of the gas tubes. For the calibration of pixel sensitivities, a procedure is developed that is valid for isotropic and anisotropic signals. The proposed procedure can save a significant amount of beamtime which has hitherto been used for calibration measurements.

## Abstract 2

A calibration procedure for small-angle neutron scattering (SANS) data is presented, where geometric effects are treated analytically and voxel sensitivities are calibrated with measurements of an arbitrary scatterer. This allows correction of the measured intensities with a single measurement, without the need to measure an isotropic scatterer at all used instrumental settings.

## 1. Introduction   

Small-angle neutron scattering (SANS) is a commonly used technique to determine the size, shape and arrangement of structures on a mesoscopic length scale in physics, chemistry, biology and materials science (Lindner *et al.*, 2000 ▸; Wang *et al.*, 2004 ▸; Petoukhov & Svergun, 2005 ▸; Davies *et al.*, 2006 ▸; Schmidt-Rohr & Chen, 2008 ▸; Fratzl, 2003 ▸; Das *et al.*, 2012 ▸; Martin *et al.*, 2013 ▸; Milde *et al.*, 2013 ▸; Gilles *et al.*, 2014 ▸; Jaksch *et al.*, 2014 ▸; Tokunaga *et al.*, 2015 ▸; Lang *et al.*, 2016 ▸).

Of course, the measured data have to be reduced in order to remove instrumental influences and obtain pure information about the sample. There are a wide range of publications on the subject of correcting SANS data. The goal is to obtain a smeared sample-dependent scattering cross section (Wignall & Bates, 1987 ▸; Strunz *et al.*, 2000 ▸; Keiderling, 2002 ▸; Ghosh *et al.*, 2000 ▸; Zemb & Lindner, 2002 ▸; Brûlet *et al.*, 2007 ▸; Zhang *et al.*, 2010 ▸; Barker & Mildner, 2015 ▸). All these methods rely on a common step, namely the calibration of the measured sample scattering signal by the signal of a well characterized isotropic scatterer, such as water, Plexiglas or vanadium, measured at the same instrumental settings as the sample.

These calibration measurements serve three purposes: (i) correction of geometric effects of the instrumental setup; (ii) correction of different detection sensitivities of different detector pixels; and (iii) calibration of the measured signal to absolute units. Unfortunately, several problems arise when performing such a calibration measurement. It is basically impossible to find a sample that scatters completely isotropically, even if incoherent, owing to inelastic (May *et al.*, 1982 ▸; Lindner *et al.*, 2000 ▸) or multiple (Copley, 1988 ▸; Shibayama *et al.*, 2005 ▸) scattering. Additionally, large detector distances require unfeasibly long measurements to achieve reasonable statistics. Therefore, the measurements are usually performed at short and medium detector distances and used for large detector distances as well, or the measurements at large detector distances are not corrected at all.

It is therefore advantageous to create a detector efficiency file at an optimal geometry for the respective standard and to transform it to the actually used geometry afterwards. This results in a significant reduction in the beamtime that has to be spent on calibration measurements. To do so, an accurate solid-angle correction has to be performed and possible anisotropic effects of the detector system must be accounted for. In this contribution, several non-trivial effects are considered that are important for detectors consisting of an array of position-sensitive ^3^He proportional counter tubes. This detector type is used in many instruments, such as SANS-1 at the Heinz Maier-Leibnitz Zentrum (MLZ) in Garching near Munich, Germany (Mühlbauer *et al.*, 2016 ▸; Heinz Maier-Leibnitz Zentrum, 2015 ▸), KWS-1 at MLZ (Feoktystov *et al.*, 2015 ▸), D22 at ILL, Grenoble, France (ILL, 2016 ▸), LOQ/SANS2D at ISIS, Oxfordshire, UK (Heenan *et al.*, 1997 ▸; Duxbury *et al.*, 2014 ▸), EQ-SANS at SNS, Tennessee, USA (Boukhalfa *et al.*, 2013 ▸), V4 at Helmholtz-Zentrum Berlin, Germany (Keiderling & Jafta, 2016 ▸), and QUOKKA at OPAL, New South Wales, Australia (Gilbert *et al.*, 2006 ▸).

The solid-angle correction for these instruments differs from that for multi-wire area detectors since the cylindrical detector tubes have an anisotropic geometry. In addition, shadowing effects arise at large angles, since multiple detection volumes intersect the trajectory of a scattered neutron. The neutron might even pass a first detector tube and be recorded in a neighbouring one, creating a parallax effect. Since the transmission of ^3^He depends on the neutron wavelength, this effect has to be understood for the whole neutron spectrum of the source. Recent SANS instruments support even more extreme geometries, such as a sideways movable detector to reach scattering angles 2θ of ∼45° (SANS-1 at MLZ). Additional corrections have to be taken into account if the detector is rotated to compensate partially for the different incident angles on the detector plane.

In the following, a procedure is proposed which solves several of the above-mentioned problems for position-sensitive ^3^He proportional counter tubes. The tools for transforming a calibration measurement to arbitrary SANS geometries are provided. This includes all available wavelengths from a cold neutron source and large scattering angles and is tested up to ∼40°. In addition, a novel procedure to correct the pixel sensitivity is introduced. Rather than relying on a completely isotropic scattering pattern, it works with any scattering pattern that is measured twice with a small lateral detector movement in between. This is advantageous, as basically no sample scatters isotropically, as mentioned above.

Several effects will be discussed that distort the measured intensity: the varying solid-angle coverage of the pixels (a change in the intensity of the order of 50%), shadowing (of the order of 25%), pixel sensitivity (of the order of 10%), scattering angle-dependent sample transmission (of the order of 3%) and finally the parallax effect (of the order of 1%).

This contribution is organized as follows. First, the geometry of the instrument will be defined. Then, four factors that influence the detected intensity will be discussed in turn: (i) the solid angle covered by the pixels; (ii) an idealized detector efficiency including shadowing and parallax effects; (iii) different pixel sensitivities; and (iv) the sample transmission for scattering at large angles. Finally, the combined influence of these factors will be demonstrated on a measured scattering pattern of vanadium.

## 2. Geometry of the instrument   

**Figure 1:**
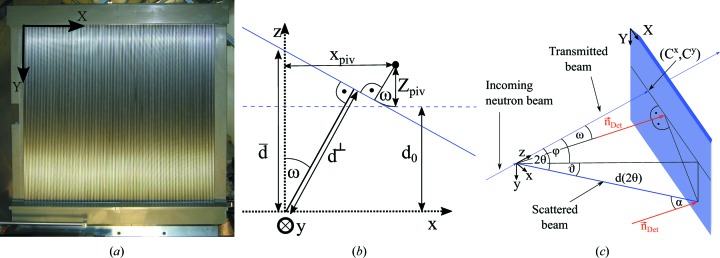
(*a)* Photograph of the SANS-1 tube detector as seen from the sample, together with a sketch of the coordinate system in detector-based coordinates. (*b*) A sketch of the sample and detector (blue) as seen from above, highlighting some of the quantities used in this contribution. The detector is shown unrotated (dashed line) and rotated (solid line). (*c*) The same as panel (*b*), seen from a different point in space. The detector plane is shown in light blue, the detector normals as red vectors and the neutron flight paths as dark blue vectors.

SANS data are usually given by an intensity matrix 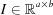, where *a* × *b* is the detector image resolution (for SANS-1 at MLZ, *a* = *b* = 128). Coordinates in this detector-based coordinate system will be denoted by uppercase vector **R** = (*X*, *Y*, *Z*) (see Fig. 1 ▸ *a*). The origin is set to the top-left pixel as seen from the sample position. *XY* is the detector plane and *Z* is the direction of the detector normal vector, all measured in pixels (px) and enumerated by 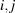 with 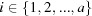 and 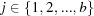. At SANS-1 at MLZ, the detector consists of tubes with an active length of slightly over 1 m, an inner diameter of 7.324 mm and a pitch of 8 mm. Each pixel has a size of 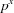 = 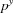 = 8 mm.

The coordinates of all pixels can also be given in a sample-based coordinate system, denoted by lowercase vector **r** = (*x*, *y*, *z*) (Figs. 1 ▸ *b* and 1 ▸ *c*). The origin is the centre of the illuminated sample volume, *z* is the direction of the incident neutron beam, and *x* and *y* are in the horizontal and vertical directions, respectively. The units of this system are metres. If the detector is not rotated (ω = 0), the two coordinate systems have parallel directions of their normal vectors, 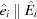, *i* = *x*, *y*, *z*.

It is customary for the detector to be able to move along the *z* direction in order to resolve a wide range of neutron scattering angles 2θ. In more flexible setups, such as that of SANS-1 at MLZ, the detector can also be moved sideways by a lateral displacement *x*_piv_ and rotated by an angle ω. For this purpose, it is mounted on a hinge that has a rotation axis parallel to *y*. The pivot point is located in the detector system at *X*_piv_ = 63.5 px and *Z*_piv_ = 9.14 px 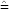 73.1 mm.

The affine transformation between the two coordinate systems is given by
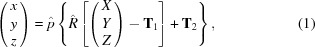
with the dimension matrix
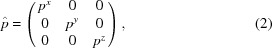
the rotation matrix around the *y* axis
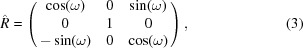
and translation vectors
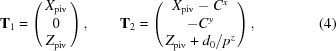
where 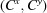 is the position of the direct beam centre on the detector. Note that gravity effects can be taken into account in the vector **T**_2_ through translation of the system’s *Y* coordinate by the corresponding offset.

The set of pixels in the sample system is defined by their centre coordinates 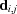 = (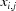, 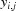, 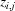) 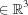 and the set of pixels in the detector system by their number 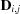 = (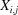, 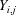, 0) 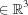, 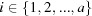, 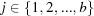. With the above transformations, the pixels can be converted from one system into the other.

Pixel 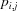 has a distance
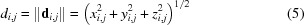
from the sample and the shortest distance between the sample and the corresponding detector tube is at 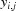 = 0, *i.e.*

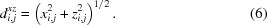
A neutron that is detected in pixel 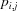 has been scattered by the sample under a scattering angle
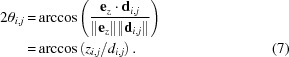
The incident angle with which the neutrons impinge on the detector can be calculated using the detector normal **n**_Det_ = [−sin(ω), 0, cos(ω)] as
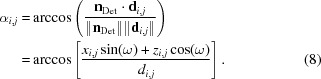
Owing to the geometry of the problem, it is beneficial to use spherical coordinates. The coordinate transformation for vector **r** is given by (Fig. 1 ▸ *c*)
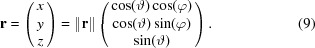
In this coordinate system, pixel 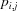 has an azimuthal angle of
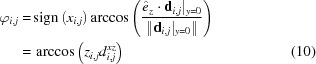
and a polar angle
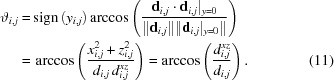
The signum function is used to orient the angles in a positive or negative direction.

If gravitational effects are taken into account, the polar angle ϑ can easily be adjusted for the loss of height of the neutrons after longer flight distances 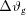 by an offset 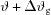.

The instrument sample-to-detector distance *d*_0_ conventionally assumes that the detector is perpendicular to the direct beam direction. For detectors that can be moved laterally and rotated by an angle ω around a pivot point, which is not necessarily in the detector plane, a different definition is needed. The distance 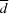 for the primary beam detected in pixel **C** is given by [as calculated from equation (1)]
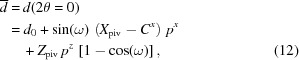
and the shortest distance from the sample-to-detector plane 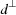 is
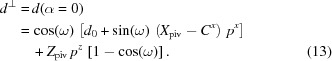



## 3. Solid-angle corrections for a flat two-dimensional detector   

It is instructive to look first at the detector as if it were a completely flat two-dimensional plane. In this case, the only effect that has to be accounted for is the variation in the solid-angle element of the different pixels. This yields the well known solid-angle correction (Grillo, 2008 ▸; He *et al.*, 2015 ▸)
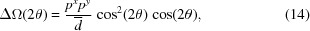
where 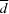 is the distance from the sample to the detector and 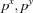 are the pixel sizes in the *x* and *y* directions, respectively. The first term represents a scaling factor taking into account that a pixel of a given size covers a smaller solid-angle element when the detector is further away from the sample. The second term, 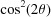, takes into account that the detector is not a spherical shell and therefore pixels with 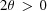 are even further away from the sample. The third term, 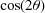, accounts for the decrease in solid-angle coverage because of the angle between the pixel surface and the scattered neutron beam.

For a detector consisting of cylindrical tubes, the 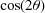 correction has to be adjusted because of the broken symmetry. While the cylinder surface tilts away from the sample as the scattering angle increases along the cylinder axis, just as it does for a flat detector, this is not the case for sideways scattered neutrons that reach a different detector tube. He *et al.* (2015 ▸) ▸ suggest a correction for the tube geometry according to
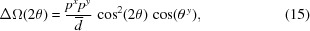
where 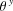 is the *y* component (*i.e.* the polar angle) of the scattering angle 2θ, *i.e.* 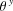 = 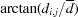.

**Figure 2:**
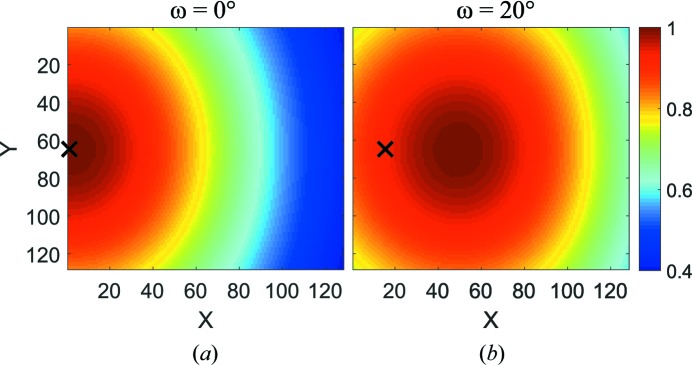
Colour plot of equation (16) for the SANS-1 beamline at MLZ, specification 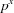 = 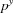 = 8 mm, 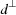 = 1.111 m, *x*_piv_ = 0.5 m, where the colour scale is normalized to 1. The beam centre is marked with a black cross. (*a*) The detector plane perpendicular to the incoming neutron beam (ω = 0) and (*b*) the detector rotated by ω = 20°.

This formula can be adjusted to a detector system rotated by an angle ω by introducing the incident detector angle from equations (8) and (11) (Fig. 1 ▸). Therewith, 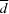 is replaced by 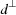, cos (2θ) by cos(α) and 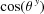 by the polar angle 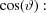


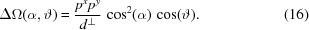
This correction is shown in Fig. 2 ▸ and is indeed the most important term of all the corrections discussed in this contribution. However, for *d* below ∼2.5 m, it becomes apparent that this treatment of a tube detector as a flat two-dimensional structure is not enough and some effects caused by the three-dimensional nature of the tubes are not accounted for. These additional corrections will be discussed below.

## 4. Idealized detector efficiency and shadowing effects in a three-dimensional detector   

At large scattering angles the detection volumes partly shadow each other, effectively reducing the illumination intensity. In the *x* direction there is a certain critical angle at which the shadowing effect starts. In the *y* direction shadowing is present for any 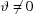. To correct for these effects, the idealized efficiency 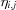 of pixel 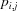 (not taking into account manufacturing differences or effects like dead time) is calculated by a numerical integration of a function of the form
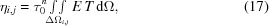
where *E* and *T* are, respectively, the absorption and transmission probability functions of a neutron with a flight trajectory through the infinitesimal detecting element dΩ. The function *E* describes the probability that a neutron is absorbed within a given pixel and *T* the probability that neighbouring pixels have not absorbed the neutron on its way. 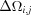 denotes the solid-angle element covered by pixel 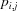. 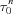 arises from the absorption function of the tube wall material, where *n* is the number of walls that the neutron intersects between the sample and ^3^He. Normally, *n* = 1, but 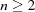 if several tubes intersect, *i.e.* if shadowing occurs.

In order to calculate *E* and *T*, the functions are parametrized in spherical coordinates (Fig. 1 ▸ *c*):
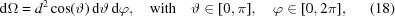
and
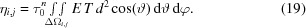
Note that *d* and 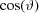 are essentially constant over a single solid-angle element 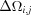, *i.e.* it is possible to write 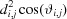 for the Jacobian determinant in the integral. The use of a cosine instead of the usual sine function is due to the definition of the coordinate system.

The transmission of ^3^He depends on the gas pressure *p*, the neutron wavelength λ and the flight path length *s* that the neutron has to travel within the gas (Masalovich, 2007 ▸):
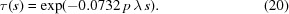
The absorption accounts for the neutrons which were not transmitted:
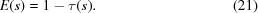
The distance *s* is a function of the exact position and angle of the incoming neutron and will be parameterized below.

In the following, the influences of the absorption probability *E* and of the shadowing and parallax effect *T* will be discussed in four stages: first, only the absorption probability without shadowing effects will be discussed at the same height as the sample, *i.e. y* = 0, before shadowing and parallax are included. Then, again without shadowing, the absorption probability will be described in the *x* and *y* directions, and finally the shadowing and parallax effects are included in these directions.

### 4.1. Absorption probability *E* in the *x* direction (*y* = 0, no shadowing)   

**Figure 3:**
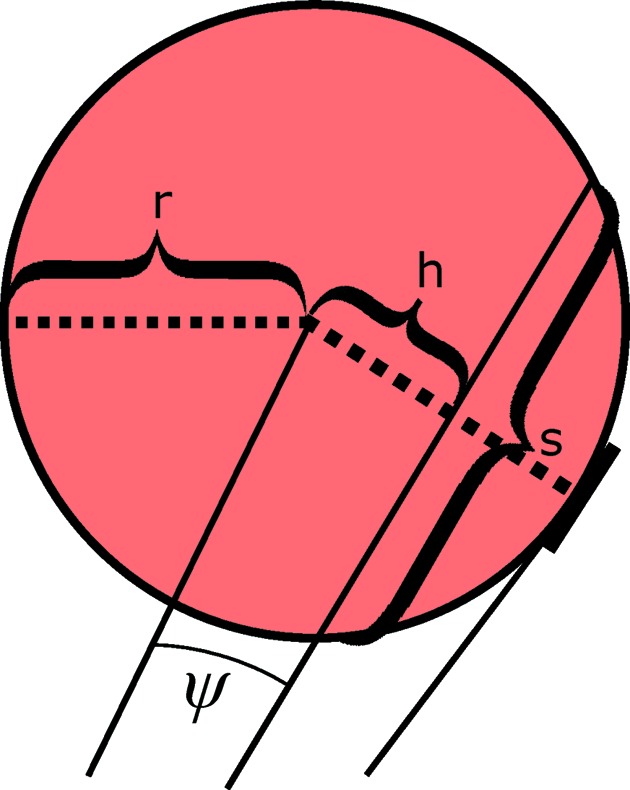
Cross section of a single detector tube of radius *r* and neutron path distance *s* at a distance *h* from the midpoint.

**Figure 4:**
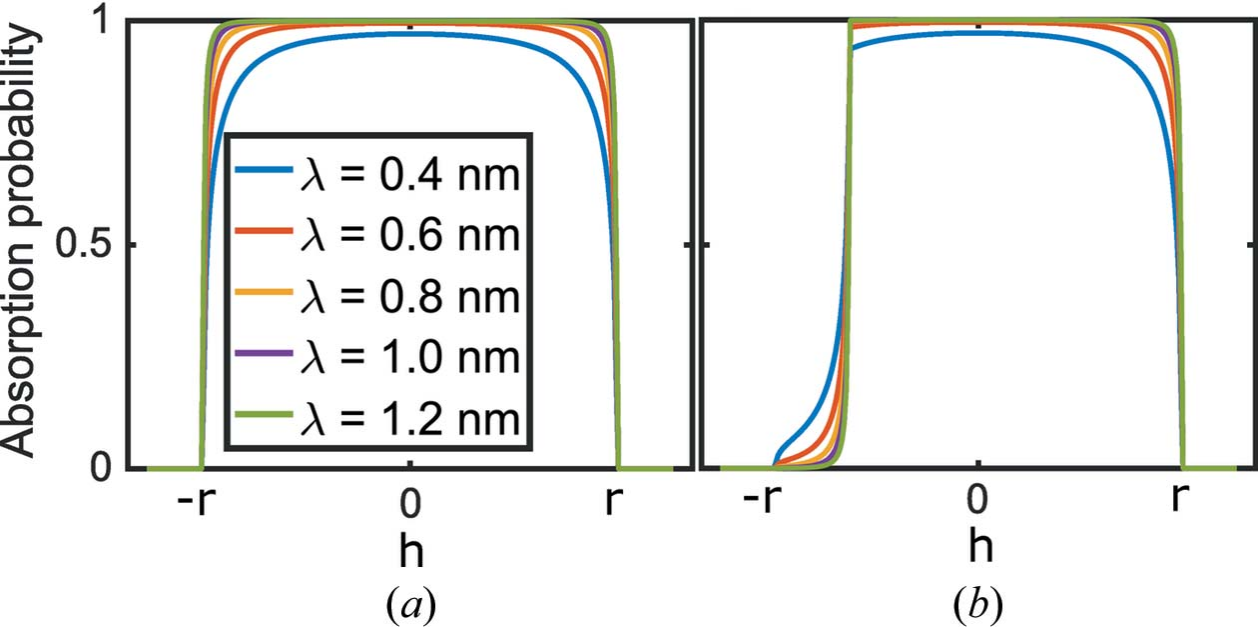
Neutron absorption probability for neutrons with several wavelengths λ impinging at a distance *h* from the centre of a detector tube of radius *r*. (*a*) Without shadowing effects, the absorption probability drops close to the edges owing to the shorter path length in the tube [equation (26)]. (*b*) A partially shadowed tube, equations (26) and (28).

At this point let (*y* = 0, ϑ = 0), *i.e.* the neutron flight trajectory is within the horizontal plane from the sample position. Hence, neutrons within this plane ‘see’ a horizontal cut through the cylindrical tube, which is a circle of radius *r*. The azimuthal opening angle ψ from the sample position to this circle is
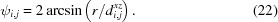
With 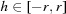 being the distance from the centre of the tube (*r* is the tube radius), the path length function *s* of the neutron depends on *h* according to (see Fig. 3 ▸)
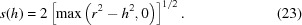
The distance *h* further depends on the azimuthal opening angle ψ and the pixel distance 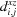 from the sample:
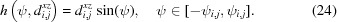
By definition of the sample-to-pixel distance 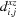 in equation (6), these opening angles are also valid for a detector system rotated by an angle ω. For pixel 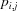, the azimuthal angle φ of the spherical coordinate system is related to the azimuthal opening angle ψ by
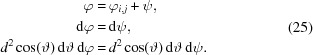
Using this substitution, the integrand of function (19) for non-shadowed pixels along the *y* = 0 plane is a function of the azimuthal opening angle ψ of pixel 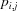:
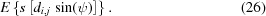
The effect is shown in Fig. 4 ▸(*a*) for different neutron wavelengths. It can be seen that the absorption probability decreases close to the left and right edges of the tube because the neutron travels through a smaller volume of the ^3^He gas.

### 4.2. Including the shadowing and parallax effect *T* in the *x* direction (*y* = 0)   

**Figure 5:**
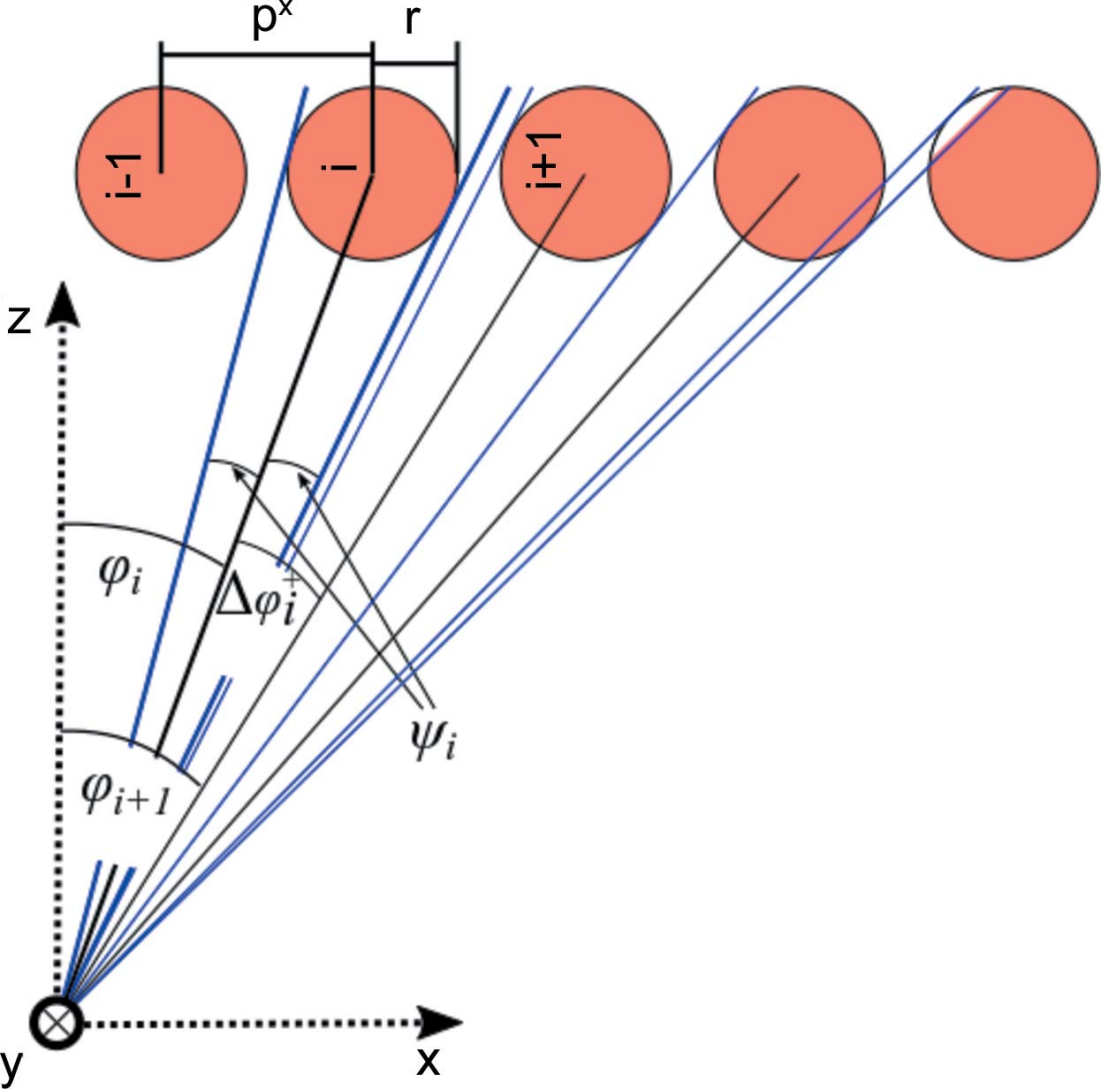
The angles 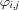 and 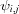. The index *j* has been omitted for readability. The tube on the right is partially shadowed by its neighbour.

Since the neutrons can hit the detector at an angle, they can cross other detector tubes before they are recorded. As a result, neutrons on the same flight trajectory can be recorded in two neighbouring pixels and a parallax effect emerges. From Fig. 5 ▸, it can be seen that the corresponding shadowing effect in the *x* direction occurs for
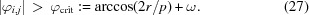
For the detector on the SANS-1 beamline at MLZ, φ_crit_ ≃ 21° + ω.

In order to take this effect into account for pixel 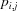 at azimuthal angle 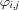, the transmission function *T* in the integrand of equation (19) is determined by the neutron flight path length through the neighbouring tubes 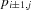 that cover the same solid-angle element 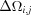 (Fig. 5 ▸). For *y* = 0 and ϑ = 0, the path length through the neighbouring tubes can be determined as a function of the azimuthal angle φ shifted by the offset of the neighbouring tube’s azimuthal angle 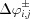 = 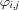 − 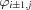. Here, a plus sign + denotes the case when the tube is shadowed on the right-hand side and a minus sign − the case on the left-hand side:
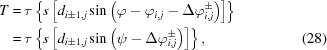
where the substitution of equation (25) was used for the azimuthal angle φ in the second step. An additional factor of the tube material transmission 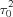 is required, since the neutrons cross the material again when leaving the shadowing tube. The neutron flux damped by the neighbouring tubes is depicted in Fig. 4 ▸(*b*).

### 4.3. Absorption probability *E* in the *x* and *y* directions (no shadowing)   

**Figure 6:**
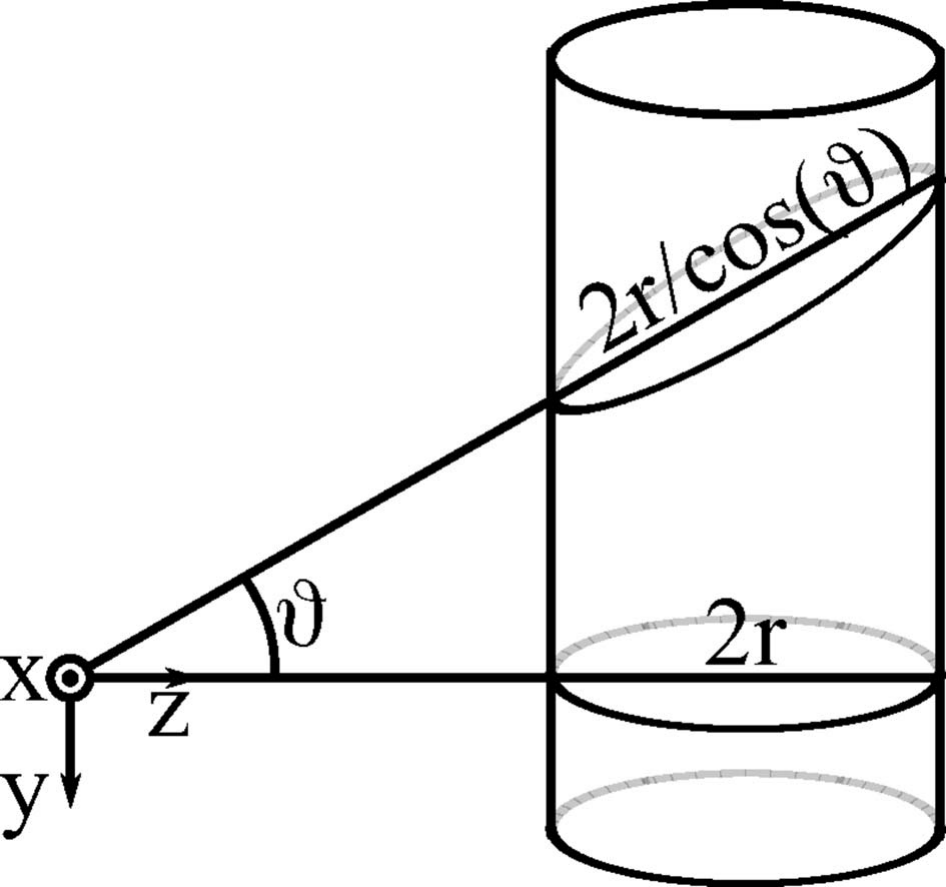
When neutrons are scattered up- or downwards, the cylinder cut is no longer a circle but an ellipse.

**Figure 7:**
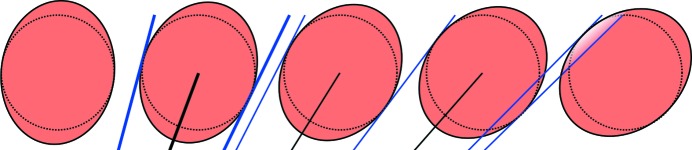
For increasing *y*, the cuts through the cylindrical tubes are ellipses.

The intersections of the horizontal plane *y* = 0 with the detector tubes are circles of radius *r*. The situation changes if the neutrons are scattered up- or downwards and impinge on the detector at a nonzero angle ϑ in the *y* direction: the mean path length is increased because the neutrons ‘see’ a skewed cut through the cylinder, which is in fact an ellipse (Fig. 6 ▸) with increasing eccentricity (Fig. 7 ▸). Hence, there is a higher probability of them being detected. For pixel 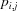, the circular cross section is deformed to an ellipse with minor axis *r* and major axis 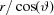 (Fig. 6 ▸) and hence *s* is increased by a factor of 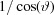 in the integrand of equation (26):
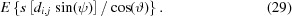
Note that 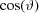 is essentially constant over the solid-angle element and it is therefore possible to use 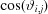.

### 4.4. Including the shadowing and parallax effect *T* in the *x* and *y* directions   

**Figure 8:**
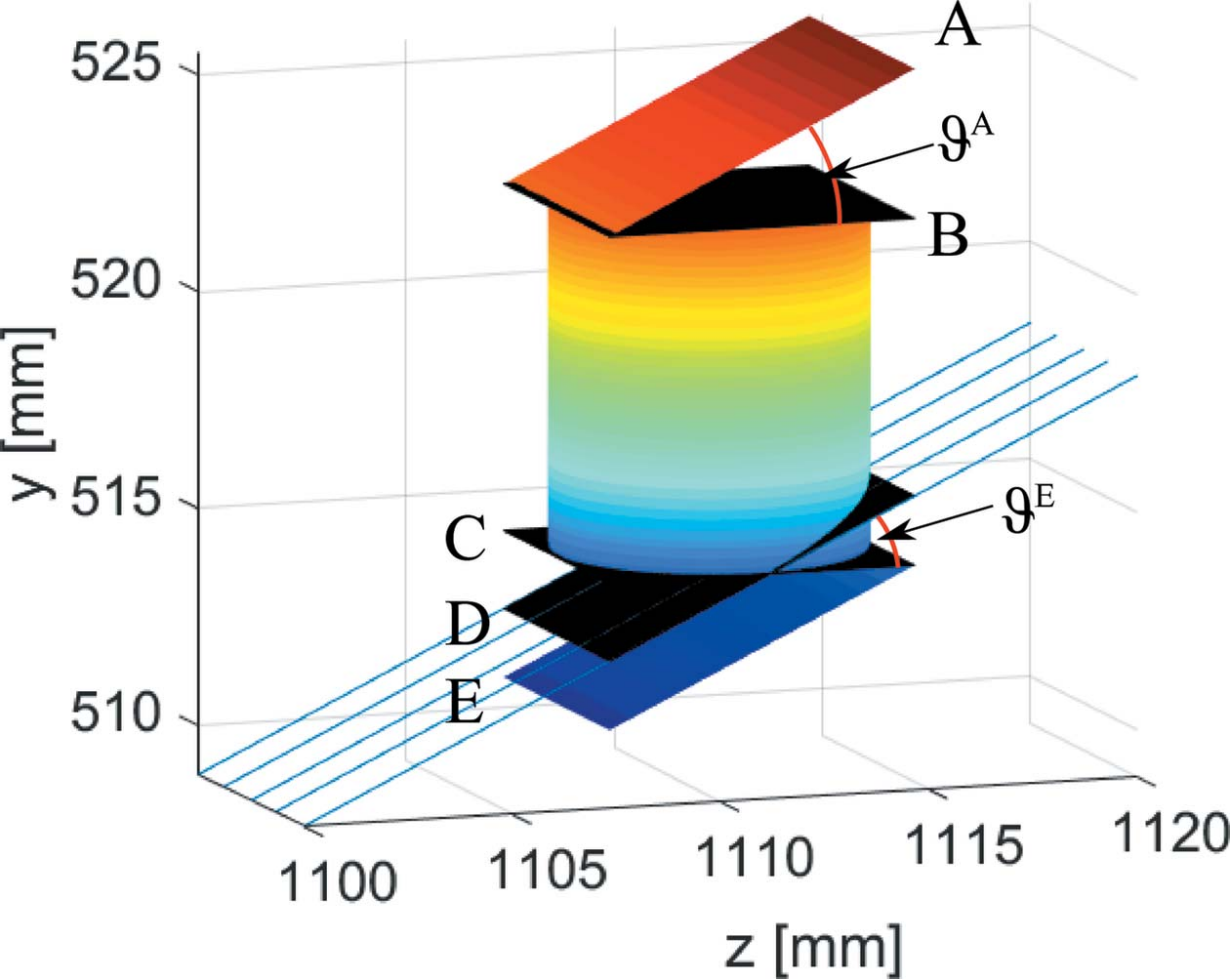
The geometry of a single voxel for ϑ = 25°. Neutrons can be partly detected in the voxel below or above, as shown for the situation with a neutron trajectory within plane *D* that intersects plane *C*.

As can be readily seen in Fig. 7 ▸, the shadowing in the *x* direction occurs for 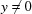 at exactly the same positions as for *y* = 0, *i.e.* the considerations from above are also valid in this case. In the *y* direction, shadowing and parallax occur for any polar angle 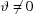. The path length function *s* has to be considered within each respective detector voxel separately. Its intersection with the upper and lower end planes of the voxel (Fig. 8 ▸) has to be calculated.

Without neglecting useful cases, it can be assumed that the neutron trajectory intersects not more than one of the confining horizontal planes. If the neutron intersects the upper or lower plane, the path length function is subdivided into *s* = *s*_in_ + *s*_out_, with the path length within the voxel *s*_in_ and that outside of it *s*_out_ (Fig. 8 ▸). This subdivision can be calculated analytically by geometric considerations (Appendix *A* [App appa]). Subsequently, similar to the *x* direction, the transmission function *T* for pixel 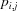 from equation (28) is further extended by the transmission function of the neutron flight path *s*_out_ in the voxel above (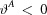) or below (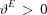):
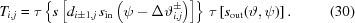
The idealized efficiency is therefore determined by an integration over the azimuthal opening angle ψ and the polar angle ϑ. The integration limits of the polar angles are determined by the confining planes *A* and *E* according to
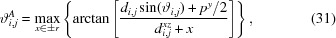


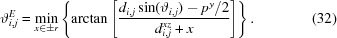
Finally, the idealized efficiency of pixel 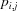 is calculated by
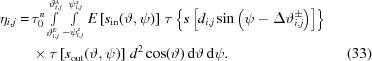
The integration can be performed numerically, for example by Gauss–Tschebyschow quadrature (Abramowitz & Stegun, 1965 ▸), with reasonable speed and very good accuracy.

**Figure 9:**
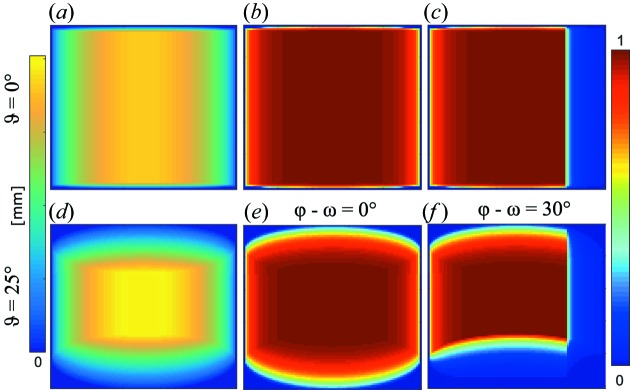
The idealized efficiency [equation (33)] of single voxels. (Top row) ϑ = 0. (*a*) A single voxel as seen from the sample position. The colour is related to the neutron flight path distance in the voxel. (*b*) The idealized efficiency of a non-shadowed voxel. (*c*) The idealized efficiency of a shadowed voxel with 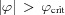. (Bottom row) ϑ = 25°. (*d*) A single voxel as seen from the sample position. (*e*) The idealized efficiency of a non-shadowed voxel. (*f*) For 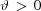, the neutron path length is truncated at the lower plane of the neighbouring voxel within the same tube. Additionally, the voxel is shadowed by the neighbouring tube for 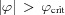.

The effect of a shadowing neighbouring tube on the right for a single pixel is shown in Figs. 9 ▸(*c*) and 9 ▸(*f*). The effect of a shadowing neighbouring voxel below in the same tube can be seen in Fig. 9 ▸(*f*).

**Figure 10:**
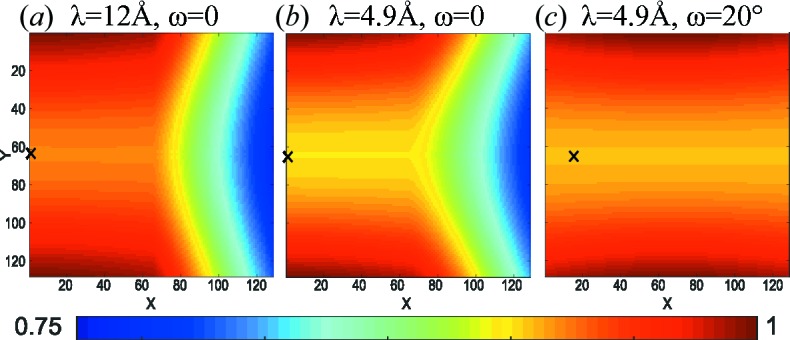
The idealized detector efficiency [equation (33)], taking into account the shadowing and the efficiency change due to the neutron path length within the cylindrical tube. (*a*) λ = 12 Å, ω = 0, (*b*) λ = 4.9 Å, ω = 0, (*c*) λ = 4.9 Å, ω = 20°, all with lateral movement of the detector *x*_piv_ = 0.5 m. The beam centre is marked with a cross. Intensities are normalized to 1.

Figs. 10 ▸(*a*) and 10 ▸(*b*) depict the idealized efficiency function due to the shadowing effects and changed neutron path length *s* in the tube for neutron wavelengths λ = 4.9 and 12 Å without any solid-angle effects. The detector is positioned at *d*_0_ = 1.111 m and *x*_piv_ = 0.5 m. The shadowing effect is strongly visible, as well as a weak efficiency increase in the *Y* direction due to the increase in the neutron flight path *s* within the tube. Fig. 10 ▸(*c*) shows the same situation but with the detector system rotated by ω = 20°. The visible effect is purely the efficiency change due to increased *s*. With this geometry the shadowing is not visible in the two-dimensional image.

## 5. Pixel sensitivity   

So far, only geometric effects have been dealt with – it has been implicitly assumed that every neutron absorbed by ^3^He corresponds to a detection event. This is not the case in practice and every pixel has an individual sensitivity ∊. The task of determining these sensitivities is often performed using an incoherent scatterer (vanadium, Plexiglas or water) which is assumed to scatter isotropically. As mentioned above, even a purely incoherent scatterer cannot be expected to result in a flat intensity distribution on the detector after all geometric corrections. In the following, a procedure is presented which does not require the sample to scatter isotropically, but instead uses two measurements of the same arbitrary sample with a displaced detector to determine the pixel sensitivity, inspired by procedures used for X-rays (Hagemann *et al.*, 2017 ▸).

For this procedure to work, it is essential to have measurements in which the intensity fluctuations due to counting statistics are much smaller than the systematic effects that are to be corrected. In the current case of sensitivity differences of the order of 10^−2^, one would aim for a relative counting error of the order of 10^−3^ or better, corresponding to at least 10^6^ neutrons per pixel. A strongly scattering sample is therefore beneficial; in the present work, glassy carbon was used.

Two measurements of the scattering of glassy carbon were performed, one with the detector further left (denoted l) and one with the detector shifted one pixel distance of 8 mm to the right (denoted r). The pixel number *i* in a given row increases from left to right, while the row number is not given for clarity. When the detector is in position r, pixel number *i* is illuminated with a true intensity *I_i_* and will register *c_i_* counts owing to its sensitivity ∊_*i*_, with statistical error (*c_i_*)^1/2^. After translating the detector left into position l, the true intensity *I_i_* is now recorded in pixel number *i* + 1:
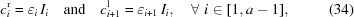
which can be combined and rearranged to
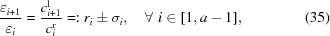
where 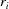 is a shorthand notation for the ratio of measured counts and σ is the propagated statistical error of the counts. In reality, the ratio of the sensitivity and the ratio of the measured counts are not identical for all pixels owing to statistical fluctuations. Instead of being equal, they should be as similar as possible, *i.e.* the aim is to minimize the quantity
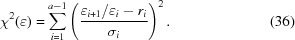
The whole set of pixel efficiencies is the solution to
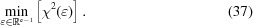
The gradient vector 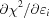 of this function can be calculated analytically, which speeds up the fitting process considerably.

The advantage of this procedure, using the ratios of neighbouring efficiencies, is that it works with arbitrary scattering patterns. Drawbacks are the necessity for very good statistics and the cancellation of a factor that is constant over the whole detector, *i.e.* an overall detector sensitivity prefactor. It follows that, with this procedure alone, the data cannot be put on an absolute scale; an additional constraint – for example the comparison of a glassy carbon measurement with the result of an X-ray measurement of the same sample – is required to fix this prefactor and obtain absolute units.

The result of the procedure presented in this section is shown in the results, using the measurements of a glassy carbon sample.

## 6. Sample effects   

There are some effects of the sample which influence the detected intensity so that it is not proportional to the scattering function. They have been described in detail elsewhere and are summarized in the following.

### 6.1. Transmission of the sample   

When neutrons are scattered to large angles (*i.e.* 2θ 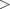 5°), the sample transmission is a function of the scattering angle 2θ and an angle-dependent transmission has to be applied. This effect can be neglected at very small angles. However, it has to be taken into account when larger scattering angles are considered.

The neutron absorption from a plate-like sample with thickness *l* is a function of the sample thickness and a material constant μ. Experimentally, the constant μ is usually approximated by a measurement of the sample transmission *T*_s_ at 2θ = 0 (Calmettes, 1999 ▸):
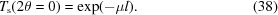
This attenuation of the transmitted beam is, on the one hand, due to simple absorption and, on the other hand, due to scattering (both coherent and incoherent) to large angles where the neutrons no longer impinge on the detector and are effectively lost.

**Figure 11:**
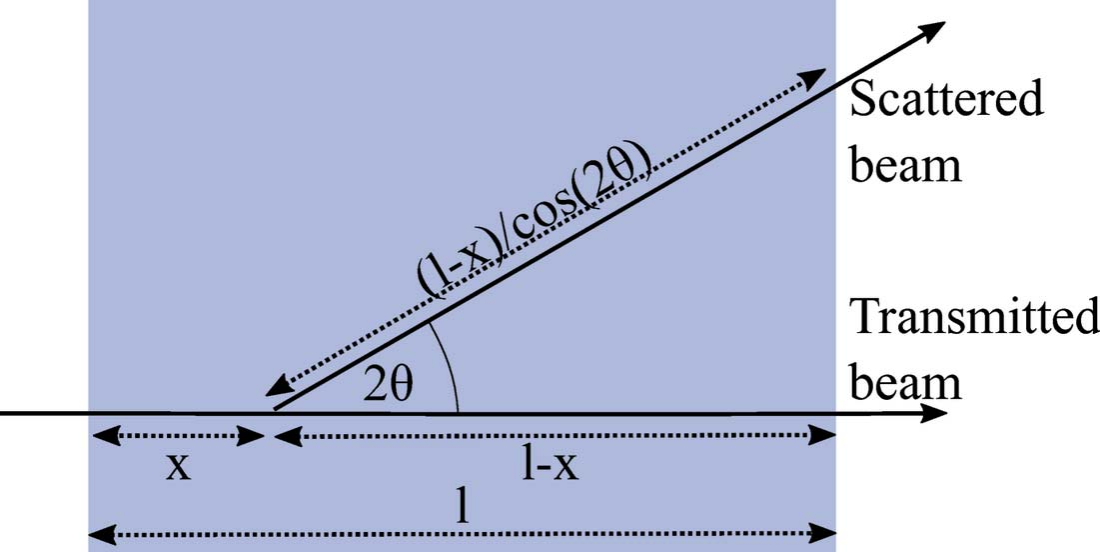
Transmission change at large angles due to the increased path length within the sample.

**Figure 12:**
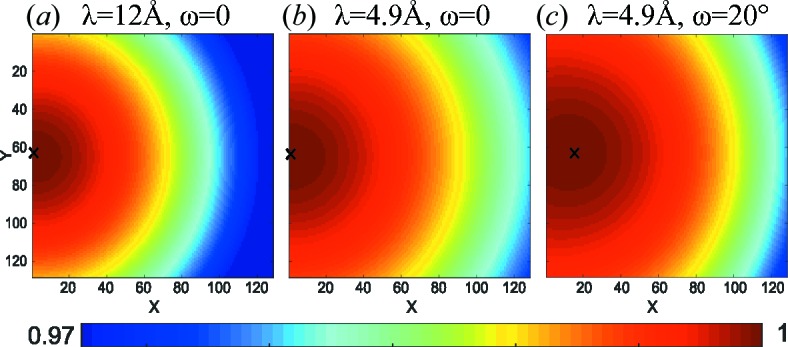
Transmission change from the vanadium sample at large angles [equation (41)]. (*a*) λ = 12 Å, ω = 0, (*b*) λ = 4.9 Å, ω = 0, (*c*) λ = 4.9 Å, ω = 20°, all with lateral movement of the detector *x*_piv_ = 0.5 m.

An additional effect that has to be taken into account is that the distance the scattered neutrons travel through a sample also depends on the scattering angle (Fig. 11 ▸). As the scattering angle increases, the flight distance increases. A neutron that is scattered after a distance *x* after entering the sample by a scattering angle 2θ traverses a distance of (Guinier, 1964 ▸)
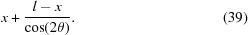
For scattering events that occur after passing a distance *x* within the sample, the neutron amplitude is attenuated by a factor of 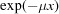 with the material-dependent value μ. The neutron amplitude that is scattered at *x* to an angle of 2θ is attenuated by an additional factor 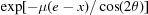. Then, the angle-dependent transmission is calculated analytically according to (Brûlet *et al.*, 2007 ▸; Grillo, 2008 ▸)
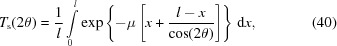
where
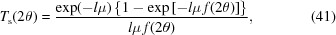
with *f*(2θ) = −1 + 1/cos (2θ). The effect is depicted in Fig. 12 ▸.

### 6.2. Inelastic scattering   

An energy transfer between the neutron and the sample causes two problems. First, the momentum transfer *Q* is calculated wrongly, and second, the detected intensity depends on the energy-dependent detection probability of the detector and a flux normalization factor. This affects both the total number of neutrons recorded by the detector and the *Q* dependence of the scattering signal, since the amount of inelastic scattering increases with *Q*. On spectrometers, the corresponding Debye–Waller factor can be accounted for; there are also correction procedures for diffractometers (Placzek, 1952 ▸) but they are less well defined.

It has been shown (Ghosh & Rennie, 1999 ▸; Ghosh *et al.*, 2000 ▸) that an energy transfer between the sample and the neutron is noticeable for many standards normally used in neutron scattering (in descending order water, Plexiglas, vanadium, glassy carbon) and has the systematic effect of increasing the neutron energy towards thermal wavelengths of ∼2 Å.

An energy transfer from the sample to the neutron requires both thermal motion in the sample and a scattering process that can transfer its energy to the neutron. Incoherent scattering probes single-particle motions and is therefore particularly susceptible to such an energy transfer. The coherent scatterer glassy carbon causes orders of magnitude less inelastic scattering than the other standards mentioned above (Barker & Mildner, 2015 ▸), which makes it particularly suitable for calibration purposes. Its only drawback is that the scattered intensity is markedly anisotropic, while the other, incoherent, standards scatter isotropically to a zeroth approximation. However, this does not pose a problem for the determination of the absolute scattering intensity, and the voxel sensitivities can also be calibrated with such an anisotropic scatterer, as shown in §5.

### 6.3. Multiple scattering   

Multiple scattering distorts the scattered intensity (Schelten & Schmatz, 1980 ▸). The calibration methods presented in this contribution are valid independent of the amount of multiple scattering in the calibration standard but do not correct for multiple scattering in the sample.

## 7. Results and discussion   

All measurements were carried out on the SANS-1 instrument at the Heinz Maier-Leibnitz Zentrum (MLZ) in Garching near Munich, Germany (Mühlbauer *et al.*, 2016 ▸; Heinz Maier-Leibnitz Zentrum, 2015 ▸). The instrument is equipped with an array of 128 position-sensitive ^3^He detectors of type Reuter-Stokes P4-0341–201 installed with a pitch of 8 mm. They have an active length of slightly more than 1 m, an inner diameter of 7.324 mm and a gas pressure *p*_He_ of 15 bar (1 bar = 100 kPa). The detector system can be moved sideways by up to 570 mm and rotated by angles of up to 21°.

**Figure 13:**
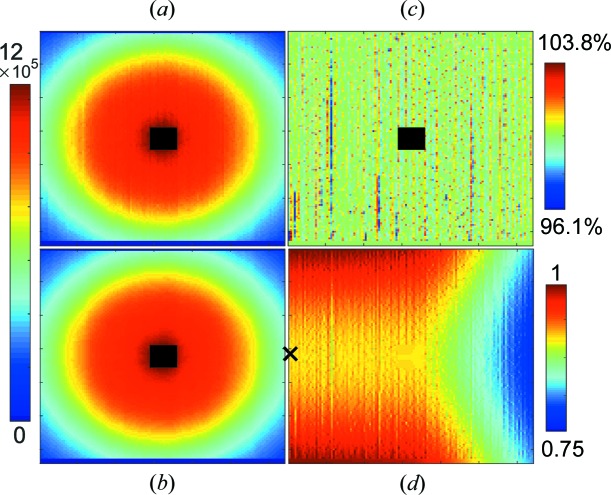
(*a*) Measurement of glassy carbon by the SANS-1 instrument at MLZ. (*b*) Glassy carbon after pixel efficiency correction. (*c*) The corresponding pixel sensitivities. Note that a total count of ∼10^6^ per pixel was obtained. (*d*) Pixel efficiency for the geometry λ = 4.9 Å, ω = 0, including the idealized detector efficiency and shadowing [equation (33)] and pixel sensitivities [equation (37)].

The determination of pixel sensitivities ∊ *via* equation (37) was performed with an established reference sample of glassy carbon (Zhang *et al.*, 2010 ▸). Its fabrication procedure is described by Moreno-Castilla *et al.* (1980 ▸). The sample had a thickness of 1 mm and was measured with a neutron wavelength of λ = 4.9 Å, a collimation of 4 m and a detector distance of *d*_0_ = 4.0 m twice: once with a lateral displacement of the detector of *x*_piv_ = 4 mm and once with *x*_piv_ = 12 mm. In order to achieve the necessary pixel statistics, the sample was measured for 10 h at both positions. The resulting sensitivity matrix is shown in Fig. 13 ▸, by itself and in combination with the idealized detector efficiency and shadowing of equation (33).

It can be seen that the procedure works very well for local sensitivity fluctuations without distorting or smearing the general shape. In particular, there is no drift across the detector from top to bottom, which might have happened since the detector can only be moved sideways and not lifted. Some features that are most probably not in the true scattering pattern have not been removed completely, in particular a vertical line of lower sensitivity around *X* = 30 and a slight distortion of the otherwise azimuthally symmetric pattern around the beamstop. However, it was decided not to enforce azimuthal symmetry and not to correct for these small effects, in order not to introduce artefacts.

Such a pixel sensitivity fluctuation map can be determined once – here at a neutron wavelength of 4.9 Å – and then used at all instrumental geometries and neutron wavelengths. The overall wavelength dependence of the detection probability is included in equation (33). The fluctuations in the pixel sensitivities are independent of wavelength (Appendix *B* [App appb]).

Using this sensitivity matrix, the rest of the procedure is demonstrated on the scattering of a vanadium sample which is a nearly perfect incoherent scatterer (Sears, 1992 ▸). For the vanadium measurements, a neutron wavelength of λ = 4.9 Å, a collimation of 6 m and a detector distance of *d*_0_ = 1.111 m were used. Additionally, offsets of *x*_piv_ = 500 mm with two distinct rotation angles ω of 0 and 20° were chosen to obtain different tube shadowing conditions. These settings require the strongest corrections of the commonly used SANS settings. The measurement time was always 30 min.

**Figure 14:**
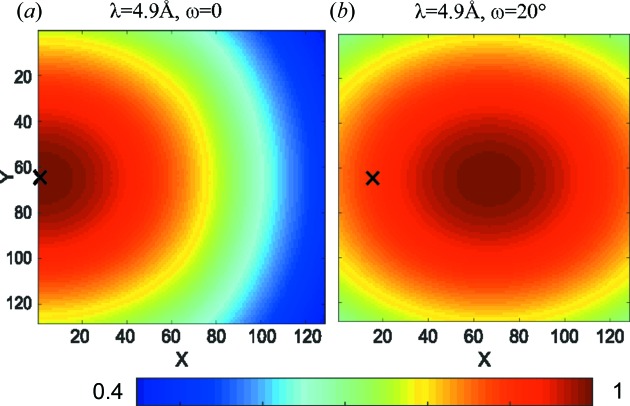
The predicted detector efficiency, including the effects from the solid-angle corrections in equation (16), the idealized detector efficiency and shadowing in equation (33), the non-ideal detection probability ∊ in equation (37), and the sample transmission correction in equation (41). (*a*) λ = 4.9 Å, ω = 0, (*b*) λ = 4.9 Å, ω = 20°, all with lateral movement of the detector *x*_piv_ = 0.5 m.

The efficiency maps for these measurement conditions were calculated by multiplying the effects from the solid-angle corrections 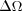 of equation (16), the idealized detector efficiency and shadowing η of equation (33), the non-ideal detection probability ∊ of equation (37), and the sample transmission correction *T*_s_ of equation (41). Fig. 14 ▸ shows the efficiency maps for two detector angles ω. Comparison with Fig. 2 ▸ shows that the solid-angle effect is the leading order correction, but the other effects introduced in this contribution have a clearly visible influence.

**Figure 15:**
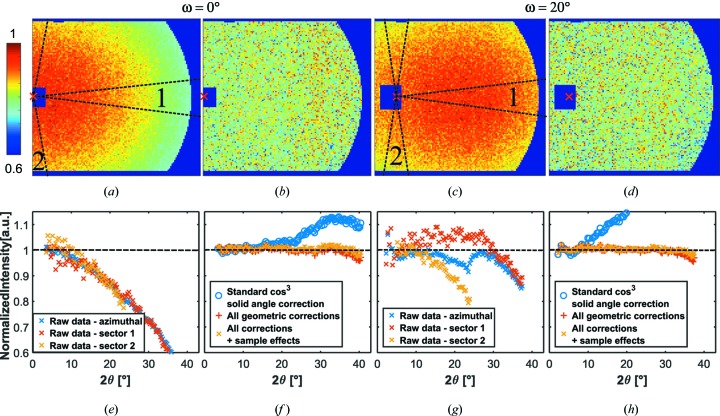
Measurement of vanadium with λ = 4.9 Å, *d* = 1.111 m, (left) with ω = 0 and (right) with rotated detector ω = 20°. (Top) Two-dimensional data, (*a*), (*c*) before and (*b*), (*d*) after applying the geometric corrections presented in this contribution. (Bottom) The corresponding azimuthally averaged data, (*e*), (*g*) before and (*f*), (*h*) after applying corrections (the classic solid-angle correction is shown for reference, and the instrument geometry effects presented in this contribution are shown for comparison, without and with sample effects). Intensities are normalized by eye to 1. The raw data were additionally evaluated in sectors, as indicated in the figure. For ω = 0, the normalized curve has a range of *I*_max_ − *I*_min_ = 1.0159 − 0.9716 = 4.4%. For ω = 20°, the range is *I*_max_ − *I*_min_ = 1.0232 − 0.9697 = 5.4%.

These efficiency maps were applied to the scattering of a vanadium sample. Figs. 15 ▸(*a*) and 15 ▸(*c*) show the results of vanadium raw data for ω = 0 and ω = 20°. Around 2θ = 0 (marked by a cross), the area of the beamstop is masked. In addition, the detector is partly overshadowed from the nose system towards the detector on the right-hand side of the pattern, which was masked as well.

With the detector orthogonal to the incoming neutron beam, ω = 0, the scattering intensity decreases by ∼50% towards the largest scattering angles owing to the solid-angle and detector effects. The rotated detector has its shortest distance to the sample nearly at its centre. For that reason, the scattering intensity decreases around that point by 10% towards the sides of the detector. Fig. 15 ▸ shows the respective scattering patterns divided by the predicted efficiencies from Fig. 14 ▸. The corrections reproduce constant scattering patterns, superimposed with statistical noise, as expected from a vanadium sample. The measurement time of the sample was not enough to resolve the sensitivity fluctuations (Fig. 13 ▸) between the pixels, but it is clear that there are no distinct systematic features visible on the two-dimensional image after the correction.

The azimuthally averaged scattering cross sections are depicted in Fig. 15 ▸. Even at the largest 2θ values of ∼40°, the procedure reproduces basically a constant. The intensity shows a very small decrease at very large 2θ values, which might be induced by inelastic scattering effects from vanadium. At ω = 20°, the averaged raw data show a kink at 2θ ≃ 23° which originates from superimposed effects in the vertical and horizontal directions. As soon as the scattering angle 2θ reaches the upper or lower boundary of the detector, the effects in the horizontal direction become suddenly more prominent, which shows the necessity for the applied anisotropic correction procedure.

## 8. Conclusions   

This study has presented a comprehensive procedure for correcting the geometric distorting effects of ^3^He counter tubes. The procedure makes it possible to create detector efficiency maps for arbitrary SANS geometries from a single measurement. It has been demonstrated that the procedure works even for extreme SANS geometries such as scattering angles of 2θ = 40° and rotated detector systems.

Additionally, a procedure for correcting the individual pixel efficiencies by taking two measurements of glassy carbon with a laterally offset detector has been demonstrated.

The procedure developed in this contribution can be easily generalized to include instrumental resolution effects. The idealized detector efficiency η is a function of λ and can therefore be smeared with a known Δλ/λ distribution function.

Performing these calibration measurements with various wavelengths at the beginning of a reactor cycle allows the calibration of all sample measurements in a very effective way, to save beam time and improve the quality of the detected data.

## 

## Figures and Tables

**Figure 16:**
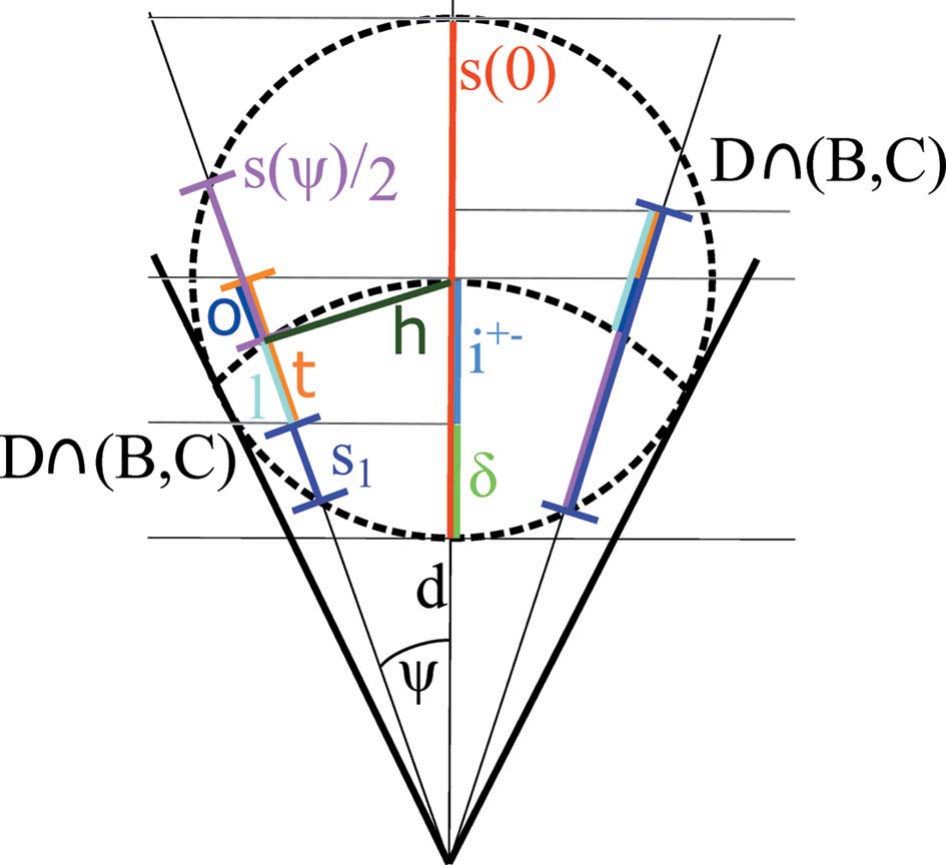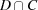 and 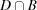 for cases where the intersection of planes is before or after the midpoint. For the line segment, *s*_1_ = *s*_out_ if 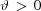 and 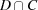, and *vice versa*.

**Figure 17:**
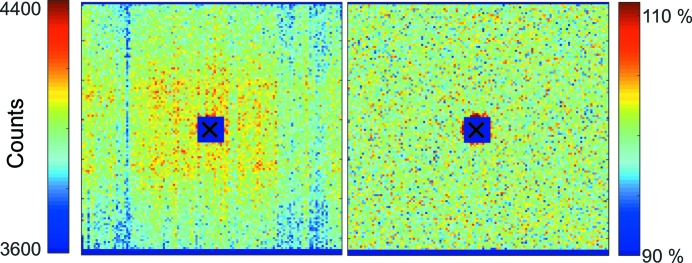
(Left) Measurement of water with a neutron wavelength of 4.9 Å at a sample-to-detector distance of 2 m with a centred detector. The tubes do not shadow each other in this geometry. Besides the general trend of higher intensity in the middle due to geometric effects, features caused by varying pixel sensitivities can be seen. A pixel-by-pixel division of this measurement by a measurement of the same sample at the same instrumental geometry, but with a neutron wavelength of 6.0 Å, is shown on the right. Apart from the statistical noise of the measurements, all effects cancel out, demonstrating their independence of the neutron wavelength.

## Appendix A Voxel limits

The intersection of a voxel’s upper or lower confining plane with the trajectory of a neutron can be calculated analytically. In the following, the geometric considerations are presented (Fig. 16 ▸). Using equations (23) and (24), the function *s* for different ψ reads

The condition that plane *D* from Fig. 8 ▸ cuts plane *B* or *C* at the boundaries of a voxel a distance *d* inside the tube and a distance  from the sample position is fulfilled if

with

where + is the condition for plane *B* and − the condition for plane *D*.

The split  =  +  is performed by the following set of equations for the case  and  (Fig. 16 ▸):

The other cases work analogously. *s*_out/in_ is decided by the angle cases  or  and whether one looks at plane *B* or *C*.

## Appendix B Wavelength dependence of pixel sensitivity

The key idea of this contribution is to separate the influence of different instrumental settings (which can be calculated) from the varying pixel sensitivities across the detector (which have to be determined experimentally). The aim is to measure the pixel sensitivities at only one instrumental setting and apply them to all measurements. Since these will most probably include measurements at other neutron wavelengths, the features in the sensitivity matrix must therefore be wavelength independent for this procedure to work.

Such an independence of neutron wavelength is to be expected, as the variations in pixel sensitivity are caused after the conversion of neutrons to charged particles in the nuclear reaction with ^3^He. The energy of these particles is independent of the neutron wavelength, which therefore does not influence the result.

This expectation was tested experimentally by measuring a sample, in this case a 1 mm thick water layer, in one instrumental geometry with two neutron wavelengths, 4.9 and 6.0 Å. While features of the pixel sensitivity are clearly visible in both measurements (Fig. 17 ▸), they cancel each other perfectly when dividing one by the other. The pixel sensitivity fluctuations are therefore not dependent on the neutron wavelength in this wavelength range.
